# Supplementary material for: 40 years of research on eating disorders in domain-specific journals: Bibliometrics, network analysis, and topic modeling
Source: PLoS One. 2022 Dec 15;17(12):e0278981. doi: 10.1371/journal.pone.0278981 (PMC9754234; doi:10.1371/journal.pone.0278981)
Supplement: S5 File — (DOCX) [file pone.0278981.s005.docx]

**Supplementary Material (S5 File)**

*Data preprocessing and text representation in Python*

As can be seen in the workflow (**Figure 1**), once network analysis was finished, a series of steps detailed below were necessary to preprocess the dataset prior to topic modeling. To represent text in data structures suitable for computational analyses we can transform it into numerical vectors. This numerical transformation is a necessary step because data mining and machine learning techniques are not properly designed to handle textual data but rather numerical data representations, or more precisely feature-based representations (see 1). Thus, a document can be represented by a list of features (terms) that can be enumerated to represent a feature space or more generally, a *vector space* (2). For example, we can represent our whole dataset as a document-term matrix. The rows represent each of our documents, the columns represent each of the words appearing in the documents (features), whereas the cells are filled with numerical values, such as the number of times a given term appears in a document. This vector space representation is called bag-of-words (BOW) because the resulting matrix is high dimensional, sparse, and this representation ignores the order as well as the syntactic and semantic information.

An alternative to BOW is using embedded models that can be represented through neural networks: a hidden layer maps words in a low-dimensional space to compute word similarity (e.g., adding vectors *Germany* and *capital* results in a vector close to *Berlin*) (3). However, embeddings tend to perform poor in domain specific words and perform well in domains where they were trained (4,5). Unfortunately, there is not an embedding model trained in the eating disorders domain. Besides its limitations, BOW representation is effective for topic discrimination (6), and works better than embeddings when the documents are very domain specific, as is the case here. Therefore, this study used a BOW approach rather than word embeddings.

Specifically, term frequency (TF) and term frequency-inverse document frequency (TF-IDF) were used for vectorization. TF vectorizer counts word occurrences within a corpus of text documents. However, some words can occur too frequently (e.g., “eating”) that can opaque more interesting terms that occurs sparsely (e.g., “objectification”). For those reasons, it is also valuable to use TF-IDF, which weights down very common terms, and the resulting weighting scheme is appropriate for further text classification and clustering. Moreover, *n-grams* were computed to capture multi-word expressions and phrases (e.g., “eating” is a unigram, whereas “eating disorder” is a bigram).

As can be intuited, some words can have different meanings depending on the context, whereas other words can have special characters due to the process of encoding text characters (glyphs) into bits, such as using Windows-1251 to encode Cyrilic characters. Moreover, some words can be irrelevant to represent a document even if they are very frequent (e.g., pronouns and articles). This can unnecessarily increase the dimensionality (number of vectors) of our vector space. For this reason, prior to the feature space construction, it was necessary to perform data *preprocessing* or, more precisely, text *normalization*.

Text mining techniques from natural language processing were used to obtain a good representation of the data (7). The steps used for text preprocessing were: tokenization (i.e., individual words), spelling correction, removing *stopwords* (i.e., pronouns, articles, etc.) (8), removing punctuations, lowercasing, and lemmatizing (i.e., transforming a word into its lemma or root: e.g., from ate to eat). For this text preprocessing process, this study relied first on the Python implementation *scispaCy*, developed by The Allen Institute for Artificial Intelligence (9). Specifically, it was used its largest pretrained model (*en_core_sci_lg*), which was trained with millions of biomedical texts and it contains a vocabulary of around 785,000 terms, and 600,000 word vectors. Second, this study used GENSIM, a Python library for natural language processing designed by RARE Technologies (2). These tools were used to perform the text preprocessing tasks described above. Once the text was normalized, it was obtained what is called a *corpus*, which is an enumerated list of all terms (features) obtained after preprocessing.

**References**

1. Brank J, Mladenić D, Grobelnik M, Milić-Frayling N. Feature selection for the classification of large document collections. J Univers Comput Sci. 2008 May;14(10):1562–96.

2. Řehůřek R, Sojka P. Software framework for topic modelling with large corpora. In: Proceedings of the LREC 2010 Workshop on New Challenges for NLP Frameworks. Valletta, Malta: ELRA; 2010. p. 45–50.

3. Zhai C, Massung S. Text data management and analysis: A practical introduction to information retrieval and text mining. New York, NY: Association for Computing Machinery; 2016.

4. Wang Y, Liu S, Afzal N, Rastegar-Mojarad M, Wang L, Shen F, et al. A comparison of word embeddings for the biomedical natural language processing. J Biomed Inform. 2018;87:12–20.

5. Kalyan KS, Sangeetha S. SECNLP: A survey of embeddings in clinical natural language processing. J Biomed Inform. 2020;101:103323.

6. Geigle C, Mei Q, Zhai C. Feature engineering for text data. In: Dong G, Liu H, editors. Feature engineering for machine learning and data analytics. Boca Raton, FL: CRC Press; 2018. p. 15–54. (Data Mining and Knowledge).

7. Nadkarni PM, Ohno-Machado L, Chapman WW. Natural language processing: An introduction. J Am Med Informatics Assoc. 2011 Sep 1;18(5):544–51.

8. Nothman J, Qin H, Yurchak R. Stop word lists in free open-source software packages. In: Proceedings of Workshop for NLP Open Source Software (NLP-OSS). Stroudsburg, PA, USA: Association for Computational Linguistics; 2018. p. 7–12.

9. Neumann M, King D, Beltagy I, Ammar W. ScispaCy: Fast and robust models for biomedical natural language processing. In: Demner-Fushman D, Cohen KB, Ananiadou S, Tsujii J, editors. Proceedings of the 18th BioNLP Workshop and Shared Task. Florence, Italy: Association for Computational Linguistics; 2019. p. 319–27.
