# Supplementary material for: 40 years of research on eating disorders in domain-specific journals: Bibliometrics, network analysis, and topic modeling
Source: PLoS One. 2022 Dec 15;17(12):e0278981. doi: 10.1371/journal.pone.0278981 (PMC9754234; doi:10.1371/journal.pone.0278981)
Supplement: Attachment — Submitted filename: Review Comments to the Author.pdf [file pone.0278981.s009.pdf]

Summary of the research and overall impression

This paper analyses documents published in leading eating disorders journals through the application of computational methods from bibliometrics and scientometrics. The author shows remarkable competence in employing a variety of methods from scientometrics and bibliometrics and proficient use of advanced packages developed for this purpose. However, there are a number of major concerns on the general aim and contribution of the article, on the selection of the corpus of documents, on the imprecise reporting of methods and results, and on the general quality of writing that prevents me to endorse its acceptance. Below I detail the observed major issues:

1. The aim of this paper is too vague (see page 3, lines 20-22/ page 7, lines 4-5). It turns to be a description of the undertaken analytic steps ("this study is aimed to analyse all scientific articles published in top peer-reviewed journals devoted to eating disorders"), instead of addressing a clear research question. Here, are just a few examples of more suitable research aims: (i) unveil the major disciplinary shifts in the literature, (ii) unveil the major contributions in the field, (iii) cross-validate previous findings or hypotheses from other narrative reviews with a different methodology.

2. The introduction is too general and it does not reach the goal to conduct the reader to the very topic of this paper. [See specific comments below]

3. A major weakness of this article is represented by the narrow bibliographic search, which limits the dataset of documents to those belonging to journals that report in their title the term "eating disorder". This choice excludes a large part of the literature on eating disorders published in interdisciplinary journals. The justifications given by the author for this choice do not seem sound. [see details below] This bias in the bibliographic search affects the overall work, limiting the strength of results.

4. In the Methods and Results important pieces of information are missing and many procedures should be explained in further detail and with a more technical language. [See specific comments below]

5. Conclusions are often redundant (information already presented in results are repeated also in this section). Interpretation of results should be better detailed and overall there are strong claims or suggestions that do not seem appropriate.

6. Generally, the whole document might be carefully revised for English spelling and phrasing. A more academic register should be employed, avoiding common language or vague expressions.

Below are more specific comments and recommended course of action by section:

Introduction

- Rephrase the aim of the paper accordingly to what detailed in point 1.
- [Point 2] The introduction starts too general. Although the covered topics are certainly interesting, they escape from the focus of this paper. We argue that there is no need to speak in general terms about the big data revolution, network analysis, text mining, or natural language processing. These concepts are too broad to be introduced in one article, whose aim is different and narrower. Indeed, this paper aims to apply these renowned techniques to review the literature on a specific research topic that is the one of "eating disorders". For this reason, I suggest reformulating the Introduction as following. Firstly, the author needs to introduce the field of "eating disorders" to the reader. Secondly, the author needs to explain the opportunity to use bibliometrics and scientometrics for the purpose of reviewing this field of research. I suggest avoiding reporting general examples about the

use of these computational techniques in unelated fields, while it could be useful to recall previous scientometric or bibliometric reviews done in the field of psychiatry, psychology, or medicine.

Methods and Materials:

Data collection

- [point 3] The author selected documents from journals titled with the term "eating disorders", merging with 8,651 documents. This choice comes unexpectedly since the standard practice for bibliographic search consists of applying the selected search syntax within the articles' title, abstract (and keywords). Using this last method to search for documents in Scopus, I retrieved 45,196 documents on 03/06/2021. The size of this corpus of documents is five times the size of the one adopted by the author and, yet, it could increase using a more comprehensive research key (i.e. the one proposed by reference n.8: "eating disorders" OR "anorexia nervosa" OR "bulimia nervosa" OR "bulimia" OR "body image"). Overall, it seems that a large part of the literature on eating disorders is excluded from the author's analysis. However, it's worth noticing that the author provided two justifications for the "narrow search": 1) they suggested the existence of "a publication bias against eating disorder articles in journals not exclusively devoted to them"; 2) they suggested that "specialized journals are expected to use a more technical lexicon that appropriately reflects the knowledge within the field". However, none of these justifications is convincing. The reference (Soh et al., 2012) used to support 1) refers to a "publication bias", speaking about the difficulty for a researcher in the field of eating disorders to be published in general psychiatry journals (in respect, for instance, to one in the field of "anxiety disorders"). This conclusion is irrelevant for the purpose of bibliographic research on eating disorders. Indeed, the author of the present article did not make any comparison between the number of citations of two different fields of research. On the contrary, within the same field documents published in interdisciplinary journals are expected to collect more global citations than those published in specialized journals. If this is true, the results from global citations could significantly differ when documents from other journals are considered. Finally, even the reference used to support the second justification (reference n.42) is inappropriate since it refers to the fact that keywords from ordinary language are often used to attract a wider audience in interdisciplinary journals. This fact is not much relevant for the discussion and, overall, we argue that also interdisciplinary journals are addressed to researchers in different fields and, thus, they keep using an appropriate technical lexicon.

  Recommended course of action:

  If the aim of the paper is to map the overall research in the field of eating disorders, it is necessary to apply the same search syntax (or a more detailed one) within the title, abstract, and keywords of documents stored in one or more databases. Otherwise, if the author wants to limit the research to specialized eating disorders journals, they need to strongly justify this choice and reframe the general aim of the paper accordingly. Even in this last case, I suggest considering the possibility to include in the dataset articles from the "Body Image" journal.

- In the Methods and Results important pieces of information are missing and many procedures should be explained in further detail and with a more technical language. [See Point 4]

  Below a list of recommended action:

  - Please, provide more information on data collection [p. 9, first paragraph]. Specify the date on which you downloaded data. Provide also the number of cited documents within your

dataset and the reason why choosing for Scopus instead of other databases (e.g. Web of Science, EBSCO, Pubmed, etc.)

  - Within the paragraph "Bibliometric Analysis and network analysis in R":
    - Please bring here the definition of "Local Citations" and "Global Citations".
    - Please, when you mention network analysis (p.9, lines 3-4-5), (i) cite the method used to compute clusters within the network; (ii) define the metrics that you will use to evaluate the results. Here I mean, for instance, the metrics that are reported in Table 3 (betweenness, closeness, PageRank).
  - The section "Data preprocessing and text representation in Python" can be shortened. In the attempt to generalize concepts, a few inaccuracies emerged in the definition of terms or in the description of the adopted procedures. For instance, embedded models (p.9, line 21) can be represented through neural networks, not as neural networks. Dictionary and corpus (line 10) are not perfect synonyms. The procedure adopted for topic modelling is not exactly based on Machine Learning (no training and test of the dataset have been performed).
  - There is no definition nor description of the performed time series analysis

Results

- Figures and Table captions are missing many pieces of information. In Table 3, the order of author might be sorted by betweenness centrality and not by PageRank, since the aim is to report the top 10 authors for centrality. It seems that Betweenness and PageRank have been confused also in the comment of results (p.16 lines 11-16).
- The text is redundant in some parts (e.g. p.16 lines 11-16), since it reports information already presented in figures of tables. Some information in the text can be inserted directly within the tables (the manual labels on page 20 can be inserted in table 4, while there are unclear passages (for instance, you may want to specify how you provided manual labels. Was the label selected based on the first keywords of the topic or did you screen the abstract of documents?)
- The interpretation of results might be improved. Overall, the provided information prevents us to go beyond the domain of descriptive analysis. I would suggest shortening the descriptive analysis (the part related to Table 1 and 2) and give more emphasis to those analyses that allow researchers to answer their research questions. Since at this moment there is no clear research question, it is difficult to understand which analysis could be the most appropriate. However, I would recommend reporting results from documents co-citation analysis (DCA) in addition to Tables 1 and 2. DCA can partially overcome the underrepresentation of recent documents (a point that was correctly highlighted in the Limitation section) by relying on metrics such as citation burst or limiting the maximum length of a citation in terms of the difference between the publication dates of the citer and the cited reference.

- **Discussion** [Point 5]
  - The Discussion provides much information already shown in the Results section (see p. 21 lines 1-15). I suggest eliminating these sessions or provide further interpretation of these results.
  - I suggest discussing all clusters within the collaboration network. [Minor point]
  - In the "Network Analysis" paragraph, the term "network" should be probably replaced with that of "cluster" from line 17 p. 22 to line 12 p.23 [Minor point]
  - Overall in the discussion, there are claims that do not seem to be supported by the results of this article (see. p.13, lines 13 – 22). In some passages, the author gives advice that goes beyond the purpose of this article (page 21, line 22 – page 22, line 4). Moreover, some results are misinterpreted or their significance is overemphasized. See, for instance, page 21, lines 16- 21. The mentioned fact the most cited documents are those related to

assessment instruments or review articles is not surprising, nor limited to the field of eating disorders. The same can be said for the growth of big research teams, which is a phenomenon that occurred across all fields of research (for a discussion of these topics see Fortunato, S., Bergstrom, C. T., Börner, K., Evans, J. A., Helbing, D., Milojević, S., ... & Barabási, A. L. (2018). Science of science. Science, 359(6379)).
