# Supplementary material for: 40 years of research on eating disorders in domain-specific journals: Bibliometrics, network analysis, and topic modeling
Source: PLoS One. 2022 Dec 15;17(12):e0278981. doi: 10.1371/journal.pone.0278981 (PMC9754234; doi:10.1371/journal.pone.0278981)
Supplement: Attachment — Submitted filename: Responses_to_Reviewers.pdf [file pone.0278981.s010.pdf]

PONE-D-21-04053
40 years of research on eating disorders: Bibliometrics, network analysis, and topic modeling
PloS One - May 10th, 2022

RESPONSES TO THE REVIEWER

REVIEWER # 1

1. *The aim of this paper is too vague (see page 3, lines 20-22/ page 7, lines 4-5). It turns to be a description of the undertaken analytic steps ("this study is aimed to analyse all scientific articles published in top peer-reviewed journals devoted to eating disorders"), instead of addressing a clear research question. Here, are just a few examples of more suitable research aims: (i) unveil the major disciplinary shifts in the literature, (ii) unveil the major contributions in the field, (iii) cross-validate previous findings or hypotheses from other narrative reviews with a different methodology.*

First, thanks to the anonymous reviewer for the fruitful suggestions to improve the article.
Second, please notice that the changes made to the original manuscripts are marked in red color to ease identification.

R1. I agree with this suggestion and now I have provided explicit research questions, as follows (from p. 9 line 21 to p. 10 line 2):

- RQ1. Which are the most cited documents in this domain-specific corpus of articles?
- RQ2. Which are the most important authors and their collaboration networks?
- RQ3. Which are the most relevant topics in this domain-specific corpus of articles?
- RQ4. How have the identified topics evolved over time (since 1981 to 2020)?

2. *The introduction is too general and it does not reach the goal to conduct the reader to the very topic of this paper. [See specific comments below]*

R2. Sorry for being too general in the introduction. I have provided below a response to each of the specific comments raised by the reviewer. So, please, see below (**R8**) specific amendments to this issue.

3. *A major weakness of this article is represented by the narrow bibliographic search, which limits the dataset of documents to those belonging to journals that report in their title the term "eating disorder". This choice excludes a large part of the literature on eating disorders published in interdisciplinary journals. The justifications given by the author for this choice do not seem sound. [see details below] This bias in the bibliographic search affects the overall work, limiting the strength of results.*

R3. Thank you very much for pointing out on this apparent issue. First, in line with the statement of research questions (**R1** above), I have changed the title of the study as follows:

40 years of research on eating disorders in domain-specific journals: Bibliometrics, network analysis, and topic modeling

Second, I apologize for not being so clear in the decision to search and retrieve solely documents from domain-specific journals on eating disorders. In fact, this sampling procedure was based on a sound rationale rather than the author's arbitrary whim, as explained in the new Introduction section (see **R8**). Now, I also have provided the procedure to select those journals (see **R9**).

On the other hand, I am aware of the literature on eating disorders in interdisciplinary journals. However, I have provided several reasons to choose field-specific core journals, as explained in the response **R9**. Moreover, including interdisciplinary journals, which is not the goal of this study, brings at least four additional difficulties: (1) the design of a search strategy, which would be chosen from a long list of many possible search variants, (2) the eventually arbitrary selection of database(s) to perform the searches, (3) the sparsity or cardinality of the term-document matrix for topic modeling, (4) the need to employ a specific methodology for interdisciplinary topic identification (see also **R9**).

4. *In the Methods and Results important pieces of information are missing and many procedures should be explained in further detail and with a more technical language. [See specific comments below]*.

R4. Thank you for pointing this out. I have improved the Methods and Results sections (see details from **R9** to **R20**).

5. Conclusions are often redundant (information already presented in results are repeated also in this section). Interpretation of results should be better detailed and overall there are strong claims or suggestions that do not seem appropriate.

R5. That's right, we apologize for it. For the sake of clarity, I removed redundant information and inappropriate claims (see details below from **R21** to **R24**).

6. Generally, the whole document might be carefully revised for English spelling and phrasing. A more academic register should be employed, avoiding common language or vague expressions.

R6. I appreciate this suggestion to improve the manuscript. The whole manuscript has been reviewed with an artificial intelligence proof-reading tool (https://writefull.ai). If the manuscript is accepted but further revision is needed to improve spelling and phrasing, I will use the ENAGO English proofreading services.

Please find below the detailed list of responses.

Introduction

- Rephrase the aim of the paper accordingly to what detailed in point 1.

R7. Thank you for the observation, I changed accordingly the title and aims of the study. Please, see **R1** and **R3** above.

- [Point 2] The introduction starts too general. Although the covered topics are certainly interesting, they escape from the focus of this paper. We argue that there is no need to speak in general terms about the big data revolution, network analysis, text mining, or natural language processing. These concepts are too broad to be introduced in one article, whose aim is different and narrower. Indeed, this paper aims to apply these renowned techniques to review the literature on a specific research topic that is the one of "eating disorders". For this reason, I suggest reformulating the Introduction as following. Firstly, the author needs to introduce the field of "eating disorders" to the reader. Secondly, the author needs to explain the opportunity to use bibliometrics and scientometrics for the purpose of reviewing this field of research. I suggest avoiding reporting general examples about the use of these computational techniques in unrelated fields, while it

could be useful to recall previous scientometric or bibliometric reviews done in the field of psychiatry, psychology, or medicine.

R8. Thank you for the observation. In line with it, the Introduction section has been entirely revised and reformulated. Now, it has a section that briefly describes the field of “eating disorders” (first paragraph). Next, it mentions what previous studies have done (second paragraph). This is followed by a description of the standard methodology used by previous studies (a query-based approach) and the limitations of it. Then, it mentions the methodology to be used in this study (selecting domain-specific journals). Next, three subsections are included (1) to explain the rationale of the methodology employed, and (2) to justify this choice. Finally, a fourth subsection was included to explain the current study, including the research questions.

At this point, I would like to mention that, since the original submission of this study (February 2021), a very similar study was published in the European Eating Disorders Review (He et al., 2022). However, the study by He et al. (2022) used a query-based approach. Therefore, I highlighted the fact that “the current study complements the work by He et al. (2022)” (p. 3 line 17). Moreover, to avoid duplication, I asked the PloS One editor in charge of this study to compare both manuscripts using some plagiarism software like Turnitin.

Reference

He, J., Kang, J., Sun, S., Cooper, M., Zickgraf, H. F., & Zhai, Y. (2022). The landscape of eating disorders research: A 40-year bibliometric analysis. European Eating Disorders Review, 30(2), 96–109. https://doi.org/10.1002/erv.2884

Methods and Materials

Data collection

- [point 3] The author selected documents from journals titled with the term “eating disorders”, merging with 8,651 documents. This choice comes unexpectedly since the standard practice for

bibliographic search consists of applying the selected search syntax within the articles' title, abstract (and keywords). Using this last method to search for documents in Scopus, I retrieved 45,196 documents on 03/06/2021. The size of this corpus of documents is five times the size of the one adopted by the author and, yet, it could increase using a more comprehensive research key (i.e. the one proposed by reference n.8: "eating disorders" OR "anorexia nervosa" OR "bulimia nervosa" OR "bulimia" OR "body image"). Overall, it seems that a large part of the literature on eating disorders is excluded from the author's analysis. However, it's worth noticing that the author provided two justifications for the "narrow search": 1) they suggested the existence of "a publication bias against eating disorder articles in journals not exclusively devoted to them"; 2) they suggested that "specialized journals are expected to use a more technical lexicon that appropriately reflects the knowledge within the field". However, none of these justifications is convincing. The reference (Soh et al., 2012) used to support 1) refers to a "publication bias", speaking about the difficulty for a researcher in the field of eating disorders to be published in general psychiatry journals (in respect, for instance, to one in the field of "anxiety disorders"). This conclusion is irrelevant for the purpose of bibliographic research on eating disorders. Indeed, the author of the present article did not make any comparison between the number of citations of two different fields of research. On the contrary, within the same field documents published in interdisciplinary journals are expected to collect more global citations than those published in specialized journals. If this is true, the results from global citations could significantly differ when documents from other journals are considered. Finally, even the reference used to support the second justification (reference n.42) is inappropriate since it refers to the fact that keywords from ordinary language are often used to attract a wider audience in interdisciplinary journals. This fact is not much relevant for the discussion and, overall, we argue that also interdisciplinary

journals are addressed to researchers in different fields and, thus, they keep using an appropriate technical lexicon.

Recommended course of action:

If the aim of the paper is to map the overall research in the field of eating disorders, it is necessary to apply the same search syntax (or a more detailed one) within the title, abstract, and keywords of documents stored in one or more databases. Otherwise, if the author wants to limit the research to specialized eating disorders journals, they need to strongly justify this choice and reframe the general aim of the paper accordingly. Even in this last case, I suggest considering the possibility to include in the dataset articles from the "Body Image" journal.

R9. I thank the reviewer for its suggestion to improve the manuscript. As stated in **R8**, the introduction has been reformulated to strongly justify the methodology chosen. Similarly, the title has been changed (**R3**) and research questions (**R1**) are provided to better reflect the aims of the study. Consequently, this study opted to analyze domain-specific journals (eating disorders journals as stated by journal's title and scope, see **R3**, and p. 11 lines 6-8). During the reformulation of the Introduction, the references mentioned by the reviewer were removed (Soh et al., 2012; Benjafield, 2019).

I also appreciate the reviewer's suggestion to include other journals such as Body Image. However, such a journal does not include eating disorders within its scope, but rather body image and human physical appearance (see https://www.sciencedirect.com/journal/body-image).

- In the Methods and Results important pieces of information are missing and many procedures should be explained in further detail and with a more technical language. [See Point 4]

Below a list of recommended action:

- Please, provide more information on data collection [p. 9, first paragraph]. Specify the date on which you downloaded data. Provide also the number of cited documents within your

dataset and the reason why choosing for Scopus instead of other databases (e.g. Web of Science, EBSCO, Pubmed, etc.).

- **R10.** The description of data collection has now more information, such as the verification of the journals' scope (p. 11 lines 6-8), the date in which the data were downloaded (p. 11 line 13), the total number of cited documents in the dataset (p. 11 line 23), and why Scopus was chosen (p. 14 lines 16). Moreover, I uploaded in the online repository (https://osf.io/5yzvd/) an additional version of the dataset (the one obtained from *biblioshiny* after preprocessing the CSV file; see p. 12 lines 2-4, 9-10). Although not mentioned in the manuscript, I would like to say that I already searched the Web of Science (WoS) database to compare it with Scopus. The main difference is that WoS includes documents not relevant to the purposes of this study, such as "book reviews". Thus, for the sake of simplicity, I preferred to use Scopus.
- Within the paragraph "Bibliometric Analysis and network analysis in R":
  - Please bring here the definition of "Local Citations" and "Global Citations".
  - **R11.** I added the definition of "Local Citations" and "Global Citations" within the paragraph "Bibliometric analysis and network analysis in R" (p. 12 lines 10-12).
  - Please, when you mention network analysis (p.9, lines 3-4-5), (i) cite the method used to compute clusters within the network; (ii) define the metrics that you will use to evaluate the results. Here I mean, for instance, the metrics that are reported in Table 3 (betweenness, closeness, PageRank).
  - **R12**. In the original manuscript I mentioned that the network analysis was performed as described by Batagelj & Cerinšek (2013) and Aria & Cuccurullo (2017) (p. 9 lines 2-3 in the original manuscript; p. 12, lines 12-13 of the new manuscript). Now, I have specified that the algorithm used for network analysis was the Louvain algorithm (p. 12 lines 15-

18). Regarding the definition of the metrics employed, when discussing the results on these metrics, it could be difficult for the readers to remember the definitions provided elsewhere. Therefore, to avoid duplicity, I provided them along with the discussion of the results (from p. 24 line 16 to p. 25 line 5).

- The section "Data preprocessing and text representation in Python" can be shortened. In the attempt to generalize concepts, a few inaccuracies emerged in the definition of terms or in the description of the adopted procedures. For instance, embedded models (p.9, line 21) can be represented through neural networks, not as neural networks. Dictionary and corpus (line 10) are not perfect synonyms. The procedure adopted for topic modelling is not exactly based on Machine Learning (no training and test of the dataset have been performed).
- **R13**. Regarding the section *"Data preprocessing and text representation in Python":* I agree with the reviewer that this section can be shortened and considering that it is something too general for readers already familiar with natural language processing, it was decided to move this entire section to the Supplementary Information (**S5** file).

  Moreover, the inaccuracies mentioned by the reviewer have been corrected (Supplementary Information S5 file: p. 1 second paragraph, p. 3 last sentence). Nevertheless, the following reviewer's statement is imprecise: "The procedure adopted for topic modelling is not exactly based on Machine Learning (no training and test of the dataset have been performed)". In fact, the code used for topic modeling was designed to perform machine learning tasks. More precisely, the Non-negative Matrix Factorization (NMF) is a well-known matrix factorization model, sometimes included among Latent Factor models and dimensionality reduction algorithms, that learns from a representation, as explained originally by Lee & Seung (1999). The machine learning model referred by the reviewer seems rather a supervised learning

model that employs a training set and a test set, such as a classification model (e.g., Support-Vector Machine, Naive Bayes classifier, etc.).

References:

- Lee, D. D. & Seung, H. S. (1999) Learning the parts of objects by non-negative matrix factorization. Nature, 401:788
- Non-negative matrix factorization. Retrieved from: https://en.wikipedia.org/wiki/Non-negative_matrix_factorization
- Support-Vector Machine. Retrieved from: https://en.wikipedia.org/wiki/Support-vector_machine

There is no definition nor description of the performed time series analysis.

- **R14.** Regarding the time series analysis, a description has been added (p. 14 lines 11-12).

Results

- Figures and Table captions are missing many pieces of information. In Table 3, the order of author might be sorted by betweenness centrality and not by PageRank, since the aim is to report the top 10 authors for centrality. It seems that Betweenness and PageRank have been confused also in the comment of results (p.16 lines 11-16).
- **R15**. First, I would like to remind that the definitions of network metrics are provided above (see **R10**). Thus, regarding the apparent confusion in the terminology, please see **R10**. Second, although "no single centrality is a consistently good ranker across networks" (Bucur, 2020, p. 1), to avoid confusion, top authors in **Table 3** are now sorted by *betweenness* centrality instead of *PageRank* (see Table 3 in p. 19).

  Reference.

- ◦ Bucur, D. (2020). Top influencers can be identified universally by combining classical centralities. *Scientific Reports*, *10*(1), 20550. https://doi.org/10.1038/s41598-020-77536-7

The text is redundant in some parts (e.g. p.16 lines 11-16), since it reports information already presented in figures of tables. Some information in the text can be inserted directly within the tables (the manual labels on page 20 can be inserted in table 4, while there are unclear passages (for instance, you may want to specify how you provided manual labels. Was the label selected based on the first keywords of the topic or did you screen the abstract of documents?)

- **R16.** Text from the original document has been removed or shortened to avoid redundancy (p. 15, lines 2-4; p. 16, lines 11-16).
- **R17.** Regarding the insertion of labels assigned to each topic within the **Table 4**, that would be ideal, but the table is already too large. In fact, it was already shortened to show only the first nine keywords out of the 10 that are shown in the output of the Python code. Regarding how those manual labels were provided, I am aware of automated ways to labeling topics (see for example, Bhatia, Lau & Baldwin, 2016). However, it would be cumbersome for the already abundant analyses of this study. Moreover, it is common practice adding labels manually in diverse analyses like factor analysis, content analysis, and topic modeling (see for example: Boyack, et al., 2011, p. 9; Ramage, et al., 2009). Consequently, the labels were manually added considering the first 10 keywords and their respective weights. The explanation in the manuscript has been slightly modified to better clarify how labels were manually added (p. 22 lines 1-2).

References.

- Bathia, S., Lau, J., & Baldwin, T. (2016). Automatic labelling of topics with neural embeddings. Retrieved from: http://arxiv.org/abs/1612.05340

- Boyack, K. W., Newman, D., Duhon, R. J., Klavans, R., Patek, M., Biberstine, J. R. … Börner, K. (2011). Clustering more than two million biomedical publications: Comparing the accuracies of nine text-based similarity approaches. *PloS One, 6*(3), 1-11.
- Ramage, et al. (2009). Topic modeling for the social sciences. Retrieved from: http://nlp.stanford.edu/dramage/papers/tmt-nips09.pdf

The interpretation of results might be improved. Overall, the provided information prevents us to go beyond the domain of descriptive analysis. I would suggest shortening the descriptive analysis (the part related to Table 1 and 2) and give more emphasis to those analyses that allow researchers to answer their research questions. Since at this moment there is no clear research question, it is difficult to understand which analysis could be the most appropriate. However, I would recommend reporting results from documents co-citation analysis (DCA) in addition to Tables 1 and 2. DCA can partially overcome the underrepresentation of recent documents (a point that was correctly highlighted in the Limitation section) by relying on metrics such as citation burst or limiting the maximum length of a citation in terms of the difference between the publication dates of the citer and the cited reference.

- **R18.** Regarding the *"shortening the descriptive analysis (the part related to Table 1 and 2)"*, that is something already done (see above **R17** about redundancy).
- **R19.** Regarding giving *"more emphasis to those analyses that allow researchers to answer their research questions"*: To avoid commenting the results in the Results section and then repeating them in the discussion (see below reviewer's Point 5), I decided to be very succinct and straightforward in the Results section, which help to reduce it. Then, in the Discussion section, I focused on the results according with the research questions (see **R1** above).

- **R20.** Regarding *"reporting results from documents co-citation analysis (DCA)".* I really appreciate this suggestion because DCA can unveil some interesting patterns in the data. However, as I just said above, the manuscript is already plenty of analyses and it could be a bit more cumbersome adding an extra layer of analysis, results (tables), and discussion. Nevertheless, now in the Discussion – Limitations section (p. 28 line 1), I inform the readers that DCA is something that can be done with the data and code from this study (Supplementary Material, https://osf.io/5yzvd/), in which it can be found an example on how to perform a co-citation analysis with the bibliographic references from the dataset. Also notice the reference below: *Co-citation analysis*, in which it is explained how to perform a co-citation analysis with the same R package used in this study (*bibliometrix*).

References.

- *Degree centrality.* https://www.sciencedirect.com/topics/computer-science/degree-centrality
- *Co-citation analysis (an example using Bibliometrix).* https://bibliometrix.org/documents/bibliometrix_Report.html#article-references-co-citation-analysis

Discussion [Point 5]

- The Discussion provides much information already shown in the Results section (see p. 21 lines 1-15). I suggest eliminating these sessions or provide further interpretation of these results.
- **R21.** In the new manuscript, the Results section has been shortened (see **R19** above), and some parts of the Discussion have been removed (e.g., p. 20, lines 21-22; p. 21 line 1, from the old manuscript).
- I suggest discussing all clusters within the collaboration network. [Minor point]
- **R22.** There were eight clusters in total and only the three most central of them have been discussed to avoid extending the length of the article. Nevertheless, the readers have the data and code to replicate the results and to perform further analyses.

- In the "Network Analysis" paragraph, the term "network" should be probably replaced with that of "cluster" from line 17 p. 22 to line 12 p.23 [Minor point]
- **R23.** Thank you very much for spotting this typo. It has been corrected in all occurrences within the Discussion section.
- Overall in the discussion, there are claims that do not seem to be supported by the results of this article (see. p.13, lines 13 – 22). In some passages, the author gives advice that goes beyond the purpose of this article (page 21, line 22 – page 22, line 4). Moreover, some results are misinterpreted or their significance is overemphasized. See, for instance, page 21, lines 16- 21. The mentioned fact the most cited documents are those related to assessment instruments or review articles is not surprising, nor limited to the field of eating disorders. The same can be said for the growth of big research teams, which is a phenomenon that occurred across all fields of research (for a discussion of these topics see Fortunato, S., Bergstrom, C. T., Börner, K., Evans, J. A., Helbing, D., Milojević, S., ... & Barabási, A. L. (2018). Science of science. Science, 359(6379)).
- **R24.** Thank you for your effort to improve this manuscript. Regarding the *"claims that do not seem to be supported by the results"* (p. 23 [not p. 13] lines 13 – 22, original manuscript), I partially agree with the reviewer. It is proposed that the methodology used here (network analysis to identify collaboration networks), "can be used along with other methodologies to establish expert finding systems in the field of eating disorders" (p. 26 lines 2-3]. This statement is something that can be performed by future studies. Then, I mention the possibilities to initiate and/or strengthen "strategic collaborations" to "promote translational research" (p. 26 line 9). This statement is in line with the discussion of the results on research collaboration. Thus, for the

sake of clarity, I decided to eliminate some text and replace some words, please see changes on the discussion, which are marked in red color to ease identification (e.g., p. 26 lines 1-10).

Regarding the results that *"are misinterpreted, or their significance is overemphasized"* (e.g., p. 21, lines 16- 21, original manuscript), and the *"advice that goes beyond the purpose of this article"* (from p. 21 line 22 to p. 22 line 4, original manuscript), I mostly agree with the reviewer. Therefore, I have removed such advice and I placed instead a more suitable implication of the results (e.g., from p. 23 line 16 to p. 24 line 8).

Finally, regarding the statement: *"the mentioned fact the most cited documents are those related to assessment instruments or review articles is not surprising, nor limited to the field of eating disorders. The same can be said for the growth of big research teams, which is a phenomenon that occurred across all fields of research (for a discussion of these topics see Fortunato, S., Bergstrom, C. T., Börner, K., Evans, J. A., Helbing, D., Milojević, S., ... & Barabási, A. L. (2018). Science of science. Science, 359(6379))."*:

I would like to say that it would be inaccurate to say that articles related to assessment instruments are the most cited in other fields, because other fields like philosophy barely use instruments. However, I agree that review articles are generally largely cited, and therefore, I have included the following statement: *"This result is not surprising considering that review articles synthesize a large number of findings"* (p. 23 lines 21-22). Regarding the statement "*The same can be said for the growth of big research teams, which is a phenomenon that occurred across all fields of research"*, I have looked for "*the growth of big research teams"* within the original manuscript and I haven't found such statement or anything related. Nevertheless, I really appreciate the reference suggested (Fortunato et al., 2018), because it has been very insightful and provides valuable information that helped me to improve the manuscript.
