# Supplementary material for: 40 years of research on eating disorders in domain-specific journals: Bibliometrics, network analysis, and topic modeling
Source: PLoS One. 2022 Dec 15;17(12):e0278981. doi: 10.1371/journal.pone.0278981 (PMC9754234; doi:10.1371/journal.pone.0278981)
Supplement: Attachment — Submitted filename: Comments_to_author.pdf [file pone.0278981.s011.pdf]

I thank the author for having revised the manuscript carefully. I will report new comments and suggested modifications below. I will mention my previous reviews and declare if the mentioned issues are resolved. Where needed, I will also report the author's answers and provide brief comments. When authors' answers are not commented on, you can assume my general satisfaction with the provided justifications and the applied modifications.

Previous major issues:

1. The aim of this paper is too vague (see page 3, lines 20-22/ page 7, lines 4-5). It turns to be a description of the undertaken analytic steps ("this study is aimed to analyse all scientific articles published in top peerreviewed journals devoted to eating disorders"), instead of addressing a clear research question.

   The author addressed this issue. The study aims are more intelligible in this new version of the manuscript.

   Minor issue: RQ4 It is unclear why the starting year is 1981 (I think this is due to the limitation of the Scopus database, starting from 1981. I would highlight this aspect in the methods and remove the dates from the aims.

2. The introduction is too general, and it does not reach the goal of conducting the reader to the very topic of this paper. [See specific comments below]

   The introduction has been improved. However, I still have major concerns about its structure and content. Specifically:

a) The attempt to justify the narrow bibliographic search, rising the problematic aspects of a query-based approach to bibliographic research, is not convincing (from line 18, p.2 to line 3, p. 5). The main argument proposed by the author is that no database is exhaustive in its coverage. You could overcome this caveat by combining the documents found in different databases. Many software designed for bibliographic analysis can simplify this process (see CiteSpace software functionalities). Secondly, the suggestion that biases are present in selecting keywords is disputable. Indeed, choosing the right entry keywords is not easy, but it is a procedure at the very basis of all systematic reviews. The technical terminology existent in ED ("eating disorders", "anorexia nervosa", "bulimia nervosa", "binge eating", etc.) makes this task more straightforward than in other domains (where the use of common words might bring to difficulties in selecting the suited literature). Therefore, the proposed argument for choosing a journal-based search is still not convincing. I would suggest focusing on how this approach can complement the findings of previous studies (lines 7-8). See also following point c.

Minor Concerns

b) An introduction to the field of eating disorders is still missing.
c) The introduction is overall too long. I would suggest not to overpass 1500/1700 words. For instance, explaining the social structure of science to justify the journal-based choice is interesting. However, I find this part too long and difficult to understand for a reader which is new to the scientometric method or unfamiliar with theories in the epistemology of science (e.g., clinical psychologists in the field of ED). Try to shorten this part and limit it to the very aim of the paper. For instance, I would suggest dedicating two lines for each journal you

selected to explain its specificity/community and audience from the perspective of the social organization of science.

3. A significant weakness of this article is represented by the narrow bibliographic search, which limits the dataset of documents to those belonging to journals that report in their title the term "eating disorder". This choice excludes a large part of the literature on eating disorders published in interdisciplinary journals. The justifications given by the author for this choice do not seem sound. [see details below] This bias in the bibliographic search affects the overall work, limiting the strength of results.

This issue is still partially valid, given the point mentioned above (2a). Moreover, the author suggests that his analysis doesn't necessarily exclude papers from other journals. I think this proposition could be correct. However, the only indicator that could potentially include documents from other journals is "global citations" (Table 2). Since you decided not to perform co-citation analysis (you are not building a co-citation network of authors or documents), you are scarcely considering papers in other journals.

4. In the Methods and Results, important pieces of information are missing, and many procedures should be explained in further detail and with a more technical language. [See specific comments below]

The methods and results improved in many parts. I have some minor suggestions since there is still some missing or unclear information.

Specifically:

p.11, line 19: explain why the starting year for your dataset is 1981.

p.12, line 20: it is not clear to me why the network included only 100 nodes (did you set this limit? Please, be precise in the number of nodes and avoid expressions like "up to/close to")

p.12, line 21: I am still convinced that a technical definition of the centrality measures might appear in the methods (in the discussion p.24-25, you can, instead, deepen their significance).

You didn't mention the cluster method to individuate the eight clusters (for instance, did you set a limited number of groups or were the 8 clusters extracted automatically?)

Table 1/Table 2 The two captions are unclear regarding the local/global differences. Please, reformulate. I would also remove (minor comment) the metrics "times cited per year" if this is computed simply as N of citation/N of years from the document publication date. This information can be misleading since the growth in citations does not usually follow a linear trend.

Figure 2 caption: Be precise in the n. of authors. Report more specific info. E.g., network modularity, colours meaning.

p. 18 lines 1-4 These considerations are more appropriate for the Discussion than for the Results session.

Table 3: Reduce decimals at 2

Cluster analysis results are not displayed. It might be interesting to visualize them. For instance, you can add a column to table 3 to mention the cluster each author belongs to, and you can create a table with the info. reported at p. 25 (e.g., a list of 5 names highest in betweenness centrality for the 3 most significant clusters).

Please, specify which text was entered to apply topic modeling (if documents abstract or the entire manuscripts. It seems to be the second option, but it might be helpful to make it explicit).

Table 4: It is not clear from the caption the weights range (maybe, it can be helpful to add a column to mention the residual weight summing all the other keywords within each topic).

The plot is not sufficient to visualize results from time series analysis. Provide further details (the added lines 11-12 are insufficient to provide a clear explanation). Specify the y-axis in the plot.

5. Conclusions are often redundant (information already presented in the results are also repeated in this section). Interpretation of results should be better detailed and overall there are strong claims or suggestions that do not seem appropriate.

Some parts of the conclusion were improved, but I still find that the interpretation of the results might go deeper. Below, I will provide suggestions for improvement.

A new major issue is the presence of a paper with the same focus of yours (He et al.,2021). Given the highly similar article by He et al.,2021, your conclusions should consider their results, tell if they are convergent or not, and propose similar/different interpretations of results (e.g., for the topic modeling).

Other suggestions, point by point:

The consideration in lines 13-15 p.22 is not a result of the analysis. Still, it is due to the dataset selection method. Maybe you want to suggest why most documents come from this journal. Is it because it is the oldest journal, or does it publish more articles yearly? Is this also the most prestigious journal in ED?

I still find the parts in which the author provides suggestions not extremely pertinent (e.g., lines 1-3 p. 264)

I want to reply to the last author's answer (R24) to clarify my point. Of course, philosophy does not include assessment instruments nor reviews in the strict conception used by psychology. What I was suggesting is that, within the field of psychology research, assessment instruments and reviews are always the most cited documents. This phenomenon is independent of the topic or the specific branch of studies (e.g., social, clinical psychology, cognitive psychology). You can undoubtedly mention this finding, but you need to elaborate more on this respect to avoid obvious claims. Just a few examples (not exhaustive) of possible discussion points: which contribution to research or diagnosis was provided by developing these assessment instruments? Do they derive from similar or different definitions of eating disorders, and why one is more cited than the others? Do they underline theoretical/methodological shifts? Do they significantly change the course of eating disorders research, and in which direction?

Recommendations in lines 16 p.23 to 3 p. 24 are not pertinent, if not better elaborated.

The mention of "Binge Eating Disorder" evolution is interesting. It would be even more interesting to understand if the citations spread before or after its inclusion in the DSM and if there are articles that, more than others, pushed its recognition.

In lines 16,17,18 p. 24, explain why these are the most cited authors. What is their contribution to the field?

I saw your answer, but I think the definition of centrality metrics is inappropriate for the Discussion, and you should move it in the methods. Here, you might explain what each indicator reveals about these authors.

When mentioning authors' clusters, it might be interesting to report the countries/institutions where they come from (you already did it for the third).

6. Generally, the whole document might be carefully revised for English spelling and phrasing. A more academic register should be employed, avoiding common language or vague expressions.

This issues was resolved.
