# Supplementary material for: 40 years of research on eating disorders in domain-specific journals: Bibliometrics, network analysis, and topic modeling
Source: PLoS One. 2022 Dec 15;17(12):e0278981. doi: 10.1371/journal.pone.0278981 (PMC9754234; doi:10.1371/journal.pone.0278981)
Supplement: Attachment — Submitted filename: responses_to_reviewer.pdf [file pone.0278981.s012.pdf]

Responses to Reviewer #1 (in red color)

I thank the author for having revised the manuscript carefully. I will report new comments and suggested modifications below. I will mention my previous reviews and declare if the mentioned issues are resolved. Where needed, I will also report the author's answers and provide brief comments. When authors' answers are not commented on, you can assume my general satisfaction with the provided justifications and the applied modifications.

Previous major issues:

1. The aim of this paper is too vague (see page 3, lines 20-22/ page 7, lines 4-5). It turns to be a description of the undertaken analytic steps ("this study is aimed to analyse all scientific articles published in top peerreviewed journals devoted to eating disorders"), instead of addressing a clear research question.

The author addressed this issue. The study aims are more intelligible in this new version of the manuscript.

Minor issue: RQ4 It is unclear why the starting year is 1981 (I think this is due to the limitation of the Scopus database, starting from 1981. I would highlight this aspect in the methods and remove the dates from the aims.

(R1) Author's Response: Thank you for the feedback. The year 1981 is the starting date of the oldest journal: the International Journal of Eating Disorders, released in Autumn (Fall) 1981: https://onlinelibrary.wiley.com/toc/1098108x/1981/1/1

To clarify this, I have made the following change in p. 6 line 12-13:

- Before: *...like the Academy of Eating Disorder, which publishes the most renowned scientific journal in the field...*
- Now: *...like the Academy of Eating Disorder, which* ***since 1981*** *publishes the most renowned scientific journal in the field…*

I also added the following sentence (p. 12 lines 8-10):

- *...It should be noted that the International Journal of Eating Disorders is the oldest of these journals, established in 1981...*

2. The introduction is too general, and it does not reach the goal of conducting the reader to the very topic of this paper. [See specific comments below]

The introduction has been improved. However, I still have major concerns about its structure and content. Specifically:

a) The attempt to justify the narrow bibliographic search, rising the problematic aspects of a query-based approach to bibliographic research, is not convincing (from line 18, p.2 to line 3, p. 5). The main argument proposed by the author is that no database is exhaustive in its coverage. You could overcome this caveat by combining the documents found in different databases. Many software designed for bibliographic analysis can simplify this process (see CiteSpace software functionalities). Secondly, the suggestion that biases are present in selecting keywords is disputable. Indeed, choosing the right entry keywords is not easy, but it is a procedure at the very basis of all systematic reviews. The technical terminology existent in ED ("eating disorders", "anorexia nervosa", "bulimia nervosa", "binge eating", etc.) makes this task more straightforward than in other domains (where the use of common words might bring to difficulties in selecting the suited literature). Therefore, the proposed argument for choosing a journal-based search is still not convincing. I would suggest focusing on how this approach can complement the findings of previous studies (lines 7-8). See also following point c.

(R2) Author's Response: I understand that the proposed approach (domain-based journals instead of a query-based search) can't be totally convincing for some readers. Therefore, I really appreciate your suggestion to overcome this issue by strengthening the focus on how the proposed approach can complement previous findings (see **R4** below).

Minor Concerns

b) An introduction to the field of eating disorders is still missing.

(R3) Author's Response: To provide the reader with a more thorough or complete introduction to the field of "eating disorders" can be a bit complicated. For example, there are several conditions with *subtypes* within the broad and diverse spectrum of eating disorders (e.g., feeding disorders, bulimia nervosa, anorexia nervosa, pica, etc.). Moreover there isn't a complete consensus in classifying eating disorders: for example, there are two standardized diagnosis criteria (DSM-5 and ICD-11; see Walsh, 2018). Nevertheless, I appreciate the reviewer's comment and I thus have provided further information to introduce the reader to eating disorders (p. 3 lines 6-9).

Walsh, B. T. (2018). Classification of eating disorders. In K. D. Brownell & B. T. Walsh (Eds.), *Eating disorders and obesity: A comprehensive handbook* (3rd ed., pp. 169-175). The Guilford Press.

c) The introduction is overall too long. I would suggest not to overpass 1500/1700 words. For instance, explaining the social structure of science to justify the journal-based choice is interesting. However, I find this part too long and difficult to understand for a reader which is new to the scientometric method or unfamiliar with theories in the epistemology of science (e.g., clinical psychologists in the field of ED). Try to shorten this part and limit it to the very aim of the paper. For instance, I would suggest dedicating two lines for each journal you selected to explain its specificity/community and audience from the perspective of the social organization of science.

(R4) Author's Response: The introduction section from the re-submitted manuscript had 2409 words (including enumerated references between parentheses). Adding an introduction to the field of eating disorders, as the reviewer suggested (see above **R3**), increased the length of the introduction to 2455 words. First, I would like to say that when I wrote this article, I had in mind three journals where to submit it. At the end, in February 2021, I decided to submit it to *PloS One* because there was already a similar article published there (Valderrama-Zurián et al., 2017), it's a multidisciplinary journal, it's open access, and as stated in the submission guidelines (https://journals.plos.org/plosone/s/submission-guidelines), "articles can be of any length". The original manuscript submitted in February 2021 had 4890 words (not including abstract, keywords, tables, figures, references), and there were 1288 words in the introduction. After receiving the first recommendations of the reviewer (June 2021), the introduction's length of the re-submitted manuscript increased from 1288 to 2409 words as stated in the first lines of this response (R4), to provide a solid rationale of the sampling procedure, which was one of the major concerns of the reviewer in the first round of review.

In any case, following the reviewer's suggestions, I cut and removed text from the introduction and now it has a length of 2002 words (not including enumerated references between parentheses). Regarding the suggestion to remove the subsection on the "social structure of science" (entitled: "*Domain-specific journals and its social structure*"): I recommend to keep it because it's just two paragraphs long (230 words long) and, as stated in the introduction (p. 5 lines 13-17), it's one of the two core arguments to justify the sampling procedure, which was the main concern of the reviewer (see R2). Moreover, in this short subsection I also included the Network Gatekeeping Theory, which is of high utility for the reader's understanding of the study. In sum, I hope that the changes I have made to reduce the introduction, balancing at the same time the requirements of the reviewer, are sufficient enough.

Valderrama-Zurián J-C, Aguilar-Moya R, Cepeda-Benito A, Melero-Fuentes D, Navarro-Moreno M-Á, Gandía-Balaguer A, et al. Productivity trends and collaboration patterns: A diachronic study in the eating disorders field. PloS One. 2017;12(8).

3. A significant weakness of this article is represented by the narrow bibliographic search, which limits the dataset of documents to those belonging to journals that report in their title the term "eating disorder". This choice excludes a large part of the literature on eating disorders published in interdisciplinary journals. The justifications given by the author for this choice do not seem sound. [see details below] This bias in the bibliographic search affects the overall work, limiting the strength of results.

> This issue is still partially valid, given the point mentioned above (2a). Moreover, the author suggests that his analysis doesn't necessarily exclude papers from other journals. I think this proposition could be correct. However, the only indicator that could potentially include documents from other journals is "global citations" (Table 2). Since you decided not to perform co-citation analysis (you are not building a co-citation network of **authors or documents**), you are scarcely considering **papers** in other journals.
>
> **(R5) Author's Response:** First, regarding this point, let me bring the response that I provided in the first round of review (see R20 of the previous response to reviewers file):
>
> > *R20. Regarding "reporting results from documents co-citation analysis (DCA)". I really appreciate this suggestion because DCA can unveil some interesting patterns in the data. However, as I just said above, the manuscript is already plenty of analyses and it could be a bit more cumbersome adding an extra layer of analysis, results (tables), and discussion. Nevertheless, now in the Discussion – Limitations section (p. 28 line 1), I inform the readers that DCA is something that can be done with the data and code from this study (Supplementary Material, https://osf.io/5yzvd/), in which it can be found an example on how to perform a co-citation analysis with the bibliographic references from the dataset. Also notice the reference below: Cocitation analysis [(an example using Bibliometrix)], in which it is explained how to perform a co-citation analysis with the same R package used in this study (bibliometrix).*
>
> At this time, I would add that, other than author/document co-citation analysis, there are several other scholarly networks that can be reported (see: Yan & Ding, 2012), such as:
>
> - *bibliographic coupling networks* (which, according to Boyack & Klavans, 2010, there has been "a resurgence in the use of bibliographic coupling that is challenging the historical preference for co-citation analysis" (p. 2390), which dates back to the early 70's)
> - *citation networks*
> - *topical networks*
> - *coauthorship networks*
> - *coword networks*

Moreover, there are different techniques, algorithms, and software, to perform co-citation analysis. For example, very recently Trujillo & Long (2022) performed Smart Local Moving (SLM) community detection algorithm to group co-cited documents and visualize them; in a prior work Gipp & Beel (2009) developed Co-citation Proximity Analysis (CPA) as a further development of document co-citation analysis; the software VOSViewer employs a clustering technique based on an algorithm for weighted networks (Yan & Ding, 2012); whilst the software *bibliometrix* used in this study allows you to select different algorithms (e.g., Leading Eigenvalues, Louvain, walktrap, spinglass, etc.); and so on.

In other words, scholarly networks and underlying techniques and algorithms are far too broad and varied that cannot be reported on in a single article.

On the other hand, co-citation analysis can be biased in some fields or knowledge niches because scientists tend to cite more frequently: (1) friends and research colleagues, (2) highly cited documents, and (3) authors with high reputation (the latter is a phenomenon called the *Matthew Effect*; Merton, 1968).

Last but not least, a co-citation analysis doesn't fit as an answer to any of the study's research questions, and that's my major concern regarding the inclusion of co-citation analyses.

Nevertheless, considering the reviewer's kind suggestion, I performed a documents co-citation analysis with the *bibliometrix* R package and selecting as main parameters the Louvain algorithm and up to 50 nodes. The results of this analysis can be found in the **Appendix** at the end of this document. The table Appendix 1 shows the top 10 documents by betweenness centrality (the complete table is available upon request), and the figure Appendix 2 shows the 46 documents (nodes) and the two clusters obtained by the analysis. Regarding the results, important and "landmark" books appear in the network (e.g., Rosenberg, 1965). Documents about well-established instruments for the assessment of eating disorders and their psychometric properties are also central in this network (e.g., Fairburn & Cooper, 1993). Finally, documents regarding clinical aspects of eating disorders (e.g. psychopathology, treatment) also appear relevant (e.g., Heatherton & Baumeister, 1991). In terms of authors, the results highlight pioneers in the field, such as Christophe G. Faiburn and David M. Garner.

In summary, although documents co-citation analyses provided interesting results, they not necessarily add substantial information to the current study, and more important, they aren't part of the current study's goals and are thus presented here at the end of this document for further information.

References

Co-citation analysis (an example using Bibliometrix). Retrieved from:

https://bibliometrix.org/documents/bibliometrix_Report.html#article-references-co-citation-analysis

Boyack, K. & Klavans, R. (2010). Co-citation analysis, bibliographic coupling, and direct citation: Which citation approach represents the research front most accurately?. *Journal of the American Society for Information Science and Technology, 61*(12), 2389-2404. https://doi.org/10.1002/asi.21419

Gipp, B. & Beel, J. (2009). Citation Proximity Analysis (CPA): A new approach for identifying related work based on co-citation analysis. In B. Larsen & J. Leta (eds.). Proceedings of the 12th International Conference on Scientometrics and Informetrics (ISSI'09) (volume 2, pp. 571–575). http://nbn-resolving.de/urn:nbn:de:bsz:352-0-285851

Merton, R. K. (1968). The Matthew Effect in science: The reward and communication systems of science are considered. *Science, 159*(3810), 56-63. https://doi.org/10.1126/science.159.3810.56

Trujillo, C. & Long T. (2018). Document co-citation analysis to enhance transdisciplinary research. *Science Advances, 4*(1), https://doi.org/10.1126/sciadv.1701130

Yan, E. & Ding, Y. (2012), Scholarly network similarities: How bibliographic coupling networks, citation networks, cocitation networks, topical networks, coauthorship networks, and coword networks relate to each other. *J Am Soc Inf Sci Tec, 63*: 1313-1326. https://doi.org/10.1002/asi.22680

4. In the Methods and Results, important pieces of information are missing, and many procedures should be explained in further detail and with a more technical language. [See specific comments below]

The methods and results improved in many parts. I have some minor suggestions since there is still some missing or unclear information.

Specifically:

p.11, line 19: explain why the starting year for your dataset is 1981.

(R6) Author's Response: Please, see the response **R1** above.

p.12, line 20: it is not clear to me why the network included only 100 nodes (did you set this limit?

Please, be precise in the number of nodes and avoid expressions like "up to/close to")

(R7) Author's Response: Thank you for pointing this out. The R package I used for authors' network analysis (bibliometrix), allows you to set several parameters, including the maximum number of nodes, which I set to up to 100 nodes.

To clarify this, I removed the expression below (p. 13 lines 12-13):

"Network parameters included: up to 100 nodes, a minimum of two edges by node, and the removal of isolated nodes."

And added the following statement (p. 13 lines 8-10; please see also **R8** below):

"It [the algorithm] works unconstrained to automatically extract a number of clusters although it requires basic network parameters as input. These network parameters were: up to 100 nodes, a minimum of two edges by node, and the removal of isolated nodes".

p.12, line 21: I am still convinced that a technical definition of the centrality measures might appear in the methods (in the discussion p.24-25, you can, instead, deepen their significance). You didn't mention the cluster method to individuate the eight clusters (for instance, did you set a limited number of groups or were the 8 clusters extracted automatically?)

(R8) Author's Response: Thank you for the suggestion. Accordingly, I have moved the technical definition of centrality measures from the Discussion section (p. 28 lines 15-16 and 20-22) to the Methods section (p. 13 lines 14-17).

Regarding *"the cluster method to individuate the eight clusters"*, it is already mentioned in the Method section (p. 13 lines 6-7): *"the Louvain algorithm for community detection (69) was used to create a collaboration network"*. This algorithm works unconstrained to detect the number of cluster, although some basic network parameters are used as input. Thus, to clarify how the eight cluster were obtained, the text has been reworded as follows:

Before (p. 13 lines 7-8): *"This algorithm identifies densely connected nodes within the network (i.e., communities) (e.g., 70)".*

Now (p. 13 lines 7-10): *"This algorithm identifies densely connected nodes within the network (i.e., communities) (e.g., 70). It works unconstrained to automatically extract a number of clusters although it requires basic network parameters as input. These network parameters were: up to 100 nodes, a minimum of two edges by node, and the removal of isolated nodes.*.

For the sake of clarity, I also reworded the paragraph in p. 18 lines 3-8 as follows:

"This collaboration network analysis identified eight clusters with 96 authors (Figure 2, see also Supplementary Material S3 File, a dataset, and S4 File, an interactive visualization in HTML and JavaScript, also available online: https://osf.io/5yzvd/): (1) red color, 4 authors; (2) blue, 15 authors; (3) green, 17 authors; (4) purple 21 authors; (5) orange, 2 authors; (6) brown, 18 authors; (7) pink, 2 authors; (8) grey, 17 authors"

Table 1/Table 2 The two captions are unclear regarding the local/global differences. Please, reformulate. I would also remove (minor comment) the metrics "times cited per year" if this is computed simply as N of citation/N of years from the document publication date. This information can be misleading since the growth in citations does not usually follow a linear trend.

(R9) Author's Response: The difference between local/global citations was already provided in the Methods section (from p. 12 line 22 to p. 13 line 2):

"Local citations (i.e., total number of citations from documents in the dataset), and global citations (i.e., citations from all documents in the whole Scopus database), were computed".

To better clarify these differences, the sentence has been changed as follows:

"Local citations (i.e., citations only from documents within the dataset), and global citations (i.e., citations made by any document from the whole Scopus database), were computed".

Regarding the captions of Table 1/ Table 2, a footnote has been added to each table as follows:

Table 1 footnote: *"Local citations are citations only from documents which come from the dataset".*

Table 2 footnote: *"Global citations are citations made by any document from the whole Scopus database".*

Moreover, the column "Total Citations" in Table 2 has been renamed "Global Citations" to avoid possible confusion.

Regarding the column "Times Cited per Year" in Table 2, it is in fact N of citation/N of years, so it was removed.

Figure 2 caption: Be precise in the n. of authors. Report more specific info. E.g., network modularity, colours meaning.

(R10) Author's Response: The Figure 2 caption has been changed as follows:

Before: *"Network of collaboration including close to 100 authors".*

Now: *"Network of collaboration including 96 authors and eight clusters".*

Regarding network modularity, I am aware of different methods (e.g., Newman, 2006), as well as software (e.g., Gephi modularity plugin) to compute it. Unfortunately, the software I used (bibliometrix) doesn't. Nevertheless, I am now providing complementary information about the results of network analysis. More precisely: the number of authors by cluster, and the clusters' color are now reported (p. 18 lines 6-8; please see also **R8** above).

Gephi modularity plugin. https://gephi.org/gephi/0.9.1/apidocs/org/gephi/statistics/plugin/Modularity.html

Newman, M. E. J. (2006). Modularity and community structure in networks. *Proceedings of the National Academy of Sciences, 103*(23), 8577-8582.

p. 18 lines 1-4 These considerations are more appropriate for the Discussion than for the Results session.

(R11) Author's Response: The text has been removed (p. 19 lines 2-4):

~~*Results from network analysis would be barely surprising for scholars familiar with eating disorder literature, because most of the authors identified in this analysis are renowned authors in the field and their contributions date back to as far as the early 1980s.*~~

Table 3: Reduce decimals at 2

(R12) Author's Response: Thank you for spotting this typo. Considering that the values of Closeness and PageRank have several decimals and start with zeros, the values were multiplied by 100 and by 10, respectively to display the values with two decimals. For example, the Closeness value 0.001831502 becomes 0.18 whereas the value 0.035365104 becomes 0.35. To explain these to readers, the following footnote has been added to Table 3:

[a] Original closeness values were multiplied by 100 to display values with only two decimals.

[b] Original PageRank values were multiplied by 10 to display values with only two decimals

Cluster analysis results are not displayed. It might be interesting to visualize them. For instance, you can add a column to table 3 to mention the cluster each author belongs to, and you can create a table with the info. reported at p. 25 (e.g., a list of 5 names highest in betweenness centrality for the 3 most significant clusters).

(R13) Author's Response: Thank you for the suggestion. As the reviewer says, not all the results of cluster analysis are displayed in Table 3. However, they are in the Supplementary Material (S3_File.csv), which displays rows for the 96 authors and columns for cluster number, betweenness, closeness, and PageRank.

Following the reviewer's suggestion, I added to Table 3 the column "Cluster", displaying the cluster number of each author. Regarding the creation of an extra Table to display, for example, *"a list of 5 names highest in betweenness centrality for the 3 most significant clusters"*, I appreciate the suggestion but it would be redundant to have such information both in the table and in the text.

Please, specify which text was entered to apply topic modeling (if documents abstract or the entire manuscripts. It seems to be the second option, but it might be helpful to make it explicit).

(R14) Author's Response: The main "text" used for topic modeling was indeed the documents' abstract, not the full text (due to copyright policies, see below). The abstract was obtained by downloading the document records from Scopus (i.e., the documents' metadata such as the title, authors, abstract, date, etc.). All metadata fields can be visualized as column names in the file named eating_disorders_literature_topic_modeling.ipynb (fourth cell with the code: *df.head()* - in the Supporting Material online: https://osf.io/89u26).

Due to copyright policies (e.g., see reference below: Elsevier Text and Data Mining), only the document records were retrieved (i.e., only document's metadata), and only the dataset obtained after preprocessing the data for topic modeling, has been shared online (dataset_preprocessed.rar: https://osf.io/rg896).

To clarify this, the following changes have been made:

p. 11 lines 15-16 and p. 12 line 2:

~~articles~~ document records

p. 12 lines 13-16:

Due to copyright, the full text of all documents was not retrieved but rather their metadata (i.e, title, author, date, abstract), whilst the dataset~~s~~ shared online (https://doi.org/10.17605/OSF.IO/5YZVD) ~~are those~~ is the one obtained after the preprocessing procedures detailed below.

Elsevier Text and Data Mining.

https://www.elsevier.com/about/policies/text-and-data-mining/text-and-data-mining-faq

Table 4: It is not clear from the caption the weights range (maybe, it can be helpful to add a column to mention the residual weight summing all the other keywords within each topic).

(R15) Author's Response: The caption of Table 4 has been changed as follows

Before:

"Results of topic modeling: First nine keywords of each topic with their respective weight"

After:

"Results of topic modeling: First nine keywords of each topic with their respective weight below each keyword".

Regarding the extra column suggested by the reviewer, Table 4 already displays only 11 columns due to issues in fitting the Table within the page's width. In any case, readers have the Supporting Material online (eating_disorders_literature_topic_modeling.ipynb: https://osf.io/89u26), in which they can visualize up to 10 keywords, and edit this line of code:

```
print_topics_udf(topics, total_topics=10, num_terms=10,
display_weights=True)
```

To display even more keywords.

The plot is not sufficient to visualize results from time series analysis. Provide further details (the added lines 11-12 are insufficient to provide a clear explanation). Specify the y- axis in the plot.
(R16) Author's Response: I understand that the reviewer refers to Figure 3. Thank you for the suggestion, I agree with it. Thus, I created a table with all values used to plot the Figure 3 (see

the new **S6** file in Supplementary Material), plus a column with the number of documents per year. The cell values in the matrix are between 0 to 11.2 (y-axis in the Figure 3), and they come from the NMF analysis to identify dominant topics. To be more precise, they are the sum of the NMF values per year and per topic. To clarify this, I changed the title of Figure 3 as follows:
Before:
"Figure 3. Time series of the ten topics found".
Now:
"Figure 3. Time series showing the topic dominance scores for the ten topics found".
I also added labels to both the y-axis (now it reads "Dominance Score") and x-axis ("Year")
Finally, I also added the following caption to Figure 3:
*"**Note.** Values in the y-axis are the sum of the weight values from the NMF analysis for topic dominance, per year and per topic. Values go from minimum 0 to maximum 11.2 (see Supplementary Material S6 file).".*

5. Conclusions are often redundant (information already presented in the results are also repeated in this section). Interpretation of results should be better detailed and overall there are strong claims or suggestions that do not seem appropriate.

Some parts of the conclusion were improved, but I still find that the interpretation of the results might go deeper. Below, I will provide suggestions for improvement. A new major issue is the presence of a paper with the same focus of yours (He et al.,2021). Given the highly similar article by He et al.,2021, your conclusions should consider their results, tell if they are convergent or not, and propose similar/different interpretations of results (e.g., for the topic modeling).

(R17) Author's Response: Thank you for your feedback to improve the discussion section. The study by He et al. (2022) has some similarities: it identifies seven topics and their trend over time, although it uses a query-based approach to obtain the articles' metadata (title, authors, abstract, etc.), and LDA instead of NMF for topic modeling; and it creates a collaboration network, although it's based on countries rather than authors. It also identifies the top journals publishing eating disorders literature, and the top authors based on well-known scientometric indexes (e.g., h-index, g-index, m-index, etc.). For the rest, most of the analyses and results from He et al. (2022) are related to geographical differences (e.g., distribution of documents by regions and countries).

Thus, regarding the two previous studies with the most similarity to the current study, the following text has been added to the manuscript (from p. 25 line 9 to p. 26 line 5):

> *"The results expand previous findings of studies that employed a query-based approach and included articles dating back as far as 1900 [13]. In particular the results expand the studies by Jinbo He et al. (2022) and Juan-Carlos Valderrama-Zurián, et al. (2017), which employed a similar methodology [1,2]. For example, He et al. (2022) created a*

collaboration network, although it was based on countries rather than authors [1]. Therefore, the results obtained here (e.g., author centrality measures, author clusters) provide a more fine grained understanding of the relevance and contribution of individual authors and their collaboration networks. Furthermore, He et al. (2022) [1] identified top authors based on traditional performance metrics (e.g., h-index), and it should be noted that there is some criticism towards their use and a claim to shift towards more responsible metrics of research excellence [87]. Then, He et al. (2022) [1] employed LDA for topic modeling, whilst this study employed NMF. Although LDA is largely used, in this study NMF outperformed LDA in interpretability, reproducibility, and as we said above, it suits better for short texts, as is the case of article abstracts used here. Finally, the top journals identified by He et al. (2022) confirmed that the five journals selected for this study are in fact among the most important in the field of eating disorders [1]. In the case of Valderrama-Zurián, et al. (2017) [2], they also focused on authors' productivity trends whereas their social network analysis was focused on network metrics such as the number of nodes and edges over time, which precludes to inspect the social network at the author level. Therefore, this study also expands on the findings of Valderrama-Zurián, et al. (2017) [2].".

Other suggestions, point by point:

The consideration in lines 13-15 p.22 is not a result of the analysis. Still, it is due to the dataset selection method. Maybe you want to suggest why most documents come from this journal. Is it because it is the oldest journal, or does it publish more articles yearly? Is this also the most prestigious journal in ED?

(R19) Author's Response: According with the suggestion, such statement can be misunderstood and therefore it has been deleted. The International Journal of Eating Disorders is in fact the oldest journal in the dataset (please see my response **R1** above). That's one of the reasons it has much more articles.

Therefore, to provide the reader with more information about the number of articles per journal and per year, I have created both a table and a Figure (files **S7** and **S8** in Supplementary Material, respectively). They are mentioned in p. 12 lines 10-11:

"The files S7 and S8 in the Supplementary Material provide the number of documents per year and per journal".

And I also added this information in the discussion section p. 26 lines 9-13:

"The top cited documents were all from the International Journal of Eating Disorders. As noted above, this journal is the oldest one (it started in 1981), and it has the largest number of articles per year, with the exception of the year 2019 when it was outperformed by the Eating and Weight Disorders journal (see S7 file and S8 file in the Supplementary Material). ~~and t~~The majority of top cited documents were related to"

I still find the parts in which the author provides suggestions not extremely pertinent (e.g., lines 1-3 p. 264)

(R20) Author's Response: The statement has been removed (p. 30 lines 3-6):

> ~~*In general, these results can help in making informed decisions about contacting experts and groups of experts in the field of eating disorders. The methodology used here can be used along with other methodologies to establish expert finding systems in the field of eating disorders (101).*~~

I want to reply to the last author's answer (R24) to clarify my point. Of course, philosophy does not include assessment instruments nor reviews in the strict conception used by psychology. What I was suggesting is that, within the field of psychology research, assessment instruments and reviews are always the most cited documents. This phenomenon is independent of the topic or the specific branch of studies (e.g., social, clinical psychology, cognitive psychology). You can undoubtedly mention this finding, but you need to elaborate more on this respect to avoid obvious claims. Just a few examples (not exhaustive) of possible discussion points: which contribution to research or diagnosis was provided by developing these assessment instruments? Do they derive from similar or different definitions of eating disorders, and why one is more cited than the others? Do they underline theoretical/methodological shifts? Do they significantly change the course of eating disorders research, and in which direction?

(R22) Author's Response: Thank you for encouraging me to improve this section. In fact, there is a gap between the use of these instruments for research and their use in clinical practice (see for example: Wade et al., 2022; Anderson & Paulosky, 2004). Being this issue so important, the discussion of these results now focus on this gap [from p. 26 line 19 to p. 27 line 6]:

> *"It should be noted, however, that in clinical practice settings the use of instruments for the diagnosis and the different phases of the treatment process is not necessarily widespread (Anderson & Paulosky, 2004). To reduce this gap, some authors suggest to provide assessment training and/or assessment guidelines for mental health professionals and general practitioners in primary health care (Hurst et al., 2020; Wade, et al., 2022). This can help obtain a comprehensive clinical assessment, particularly of individuals with higher risk such as young adolescents with restrictive Anorexia Nervosa (Society for Adolescent Health and Medicine, 2015). The instruments mentioned above are reliable measures, and they could be used online for a quick screening or session by session for ongoing monitoring, although further research is necessary (e.g., Chan & Leung, 2015; Sandberg & Erford, 2013; Tatham et al. 2015)".*

- *Anderson & Paulosky (2004). A survey of the use of assessment instruments by eating disorder professionals in clinical practice. Eating and Weight Disorders, 9(3), pp. 238-241. 10.1007/BF03325075*

- *Chan, C. W. & Leung, S. F. (2015). Validation of the Eating Disorder Examination Questionnaire: An online version. Journal of Human Nutrition and Dietetics, 28(6), 659-665. https://doi.org/10.1111/jhn.12275*
- *Hurst, K., Heruc, G., Thornton, C. et al. ANZAED practice and training standards for mental health professionals providing eating disorder treatment. J Eat Disord 8, 58 (2020). https://doi.org/10.1186/s40337-020-00333-0*
- *Sandberg, K. and Erford, B.T. (2013), Choosing Assessment Instruments for Bulimia Practice and Outcome Research. Journal of Counseling & Development, 91: 367-379. https://doi.org/10.1002/j.1556-6676.2013.00107.x*
- *Tatham, M. et al. (2015). Development, psychometric properties and preliminary clinical validation of a brief, session-by-session measure of eating disorder cognitions and behaviors: The ED-15. International Journal of Eating Disorders, 48(7), 1005-1015. https://doi.org/10.1002/eat.22430*
- *The Society for Adolescent Health and Medicine (2015). Position paper of the Society for Adolescent Health and Medicine: Medical management of restrictive eating disorders in adolescents and young adults. Journal of Adolescent Health, 56(1), 121-125. https://doi.org/10.1016/j.jadohealth.2014.10.259*
- *Wade, T. D., Johnson, C., Cadman, K., & Cook, L. (2022). Turning eating disorders screening in primary practice into treatment: A clinical practice approach. International Journal of Eating Disorders, 1– 5. https://doi.org/10.1002/eat.23732*

Recommendations in lines 16 p.23 to 3 p. 24 are not pertinent, if not better elaborated.

(R23) Author's Response: Those recommendations were removed (p. 27 lines 13-22):

> ~~*In general, these results indicate that much of the research on eating disorders in the last decades has been based on self-reports. Self-reports are known to have limitations such as social desirability and response bias (e.g., 84). Future studies should consider novel instruments, methodologies, and frameworks such as ecological momentary assessment or complex systems (83,85,86). On the other hand, review studies on the epidemiology, course, and outcome of eating disorders are highly cited because they provide key information on eating disorders. This result is not surprising considering that review articles synthesize a large number of findings (8,9). A possible implication of this result can be the integration of the results of high-quality review articles in the education and training of health professionals who care for people with eating disorders (87,88).*~~

The mention of "Binge Eating Disorder" evolution is interesting. It would be even more interesting to understand if the citations spread before or after its inclusion in the DSM and if there are articles that, more than others, pushed its recognition.

(R24) Author's Response: I am thankful for the suggestion to further explore potentially interesting results associated with this finding. However, it would require to perform analyses beyond the scope of those already done.

In lines 16,17,18 p. 24, explain why these are the most cited authors. What is their contribution to the field?.

I saw your answer, but I think the definition of centrality metrics is inappropriate for the Discussion, and you should move it in the methods. Here, you might explain what each indicator reveals about these authors.

When mentioning authors' clusters, it might be interesting to report the countries/institutions where they come from (you already did it for the third).

(R25) Author's Response: Regarding p. 24 lines 16-18, please let me correct: I didn't indicated that those are the *most cited* authors. I indicated that *"The author with the largest betweenness centrality was Ross D Crosby, followed by James E Mitchell which has the largest value in PageRank"* (now in p. 28 lines 13-15). Then, I provided an explanation of what these results mean (in terms of "betweenness centrality") (p. 28 lines 17-20).

Regarding the suggestion to move the definition of centrality metrics from the discussion to the methods section, I followed your suggestion and I did it (please see **R8** above).

Regarding authors' institution/country, thank you for the suggestion. I added such information for each author.

6. Generally, the whole document might be carefully revised for English spelling and phrasing. A more academic register should be employed, avoiding common language or vague expressions.

This issues was resolved.

APPENDIX

Appendix 1. Documents co-citation analysis: Top 10 most central documents according to betweenness centrality.*

#	Reference	Node label	Cluster	Betweenness
1	Garner, D. M. & Wooley, S. (1991). Confronting the failure of behavioral and dietary treatments for obesity. Clinical Psychology Review, 11(6), 729-780. https://doi.org/10.1016/0272-7358(91)90128-H	garner d.m. 1991	2	196.24
2	Fairburn, C. G., & Cooper, Z. (1993). The Eating Disorder Examination (12th edition). In C. G. Fairburn & G. T. Wilson (Eds.), Binge eating: Nature,	fairburn c.g. 1993	1	116.26

	assessment, and treatment (pp. 317–360). Guilford Press.			
3	Fairburn, C.G. and Beglin, S.J. (1994), Assessment of eating disorders: Interview or self-report questionnaire?. Int. J. Eat. Disord., 16: 363-370. https://doi.org/10.1002/1098-108X(199412)16:4<363::AID-EAT2260160405>3.0.CO;2-#	fairburn c.g. 1994	1	109.14
4	Garner, D. M., Olmsted, M. P., Bohr, Y., & Garfinkel, P. E. (1982). The Eating Attitudes Test: Psychometric features and clinical correlates. Psychological Medicine, 12(4), 871–878. https://doi.org/10.1017/S0033291700049163	garner d.m. 1982	2	74.91
5	Rosenberg, M. (1965). Society and the adolescent self-image. Princeton University Press	rosenberg m. 1965	2	58.28
6	Heatherton, T. F., & Baumeister, R. F. (1991). Binge eating as escape from self-awareness. Psychological Bulletin, 110(1), 86–108. https://doi.org/10.1037/0033-2909.110.1.86	heatherton t.f. 1991	1	54.50
7	Garner, D. M., Olmsted, M. P., & Garfinkel, P. E. (1983). Does Anorexia Nervosa occur on a continuum? Subgroups of weight-preoccupied women and their relationship to Anorexia Nervosa. International Journal of Eating Disorders, 2(4), 11-20. https://doi.org/10.1002/1098-108X(198322)2:4<11::AID-EAT2260020403>3.0.CO;2-W	garner d.m. 1983-2	2	52.32
8	Garner, D. M., & Garfinkel, P. E. (1979). The Eating Attitudes Test: An index of the symptoms of anorexia nervosa. Psychological Medicine, 9(2), 273–279. https://doi.org/10.1017/S0033291700030762	garner d.m. 1979	2	52.10
9	Beck, A. T., Ward, C. H., Mendelson, M., Mock, J., & Erbaugh, J. (1961). An inventory for measuring depression. Archives of General Psychiatry, 4, 561–571. https://doi.org/10.1001/archpsyc.1961.01710120031004	beck a.t. 1961	2	44.37
10	Bruch, H. (1973). Eating Disorders: Obesity, Anorexia Nervosa, And The Person Within. Basic Books	bruch h. 1973	2	31.74

Note. *Analysis performed with *bibliometrix* R package. The Louvain algorithm was employed for clustering. A total of 46 documents and two clusters were retrieved by the analysis.

Appendix 2. Figure showing the results of documents co-citation analysis.*

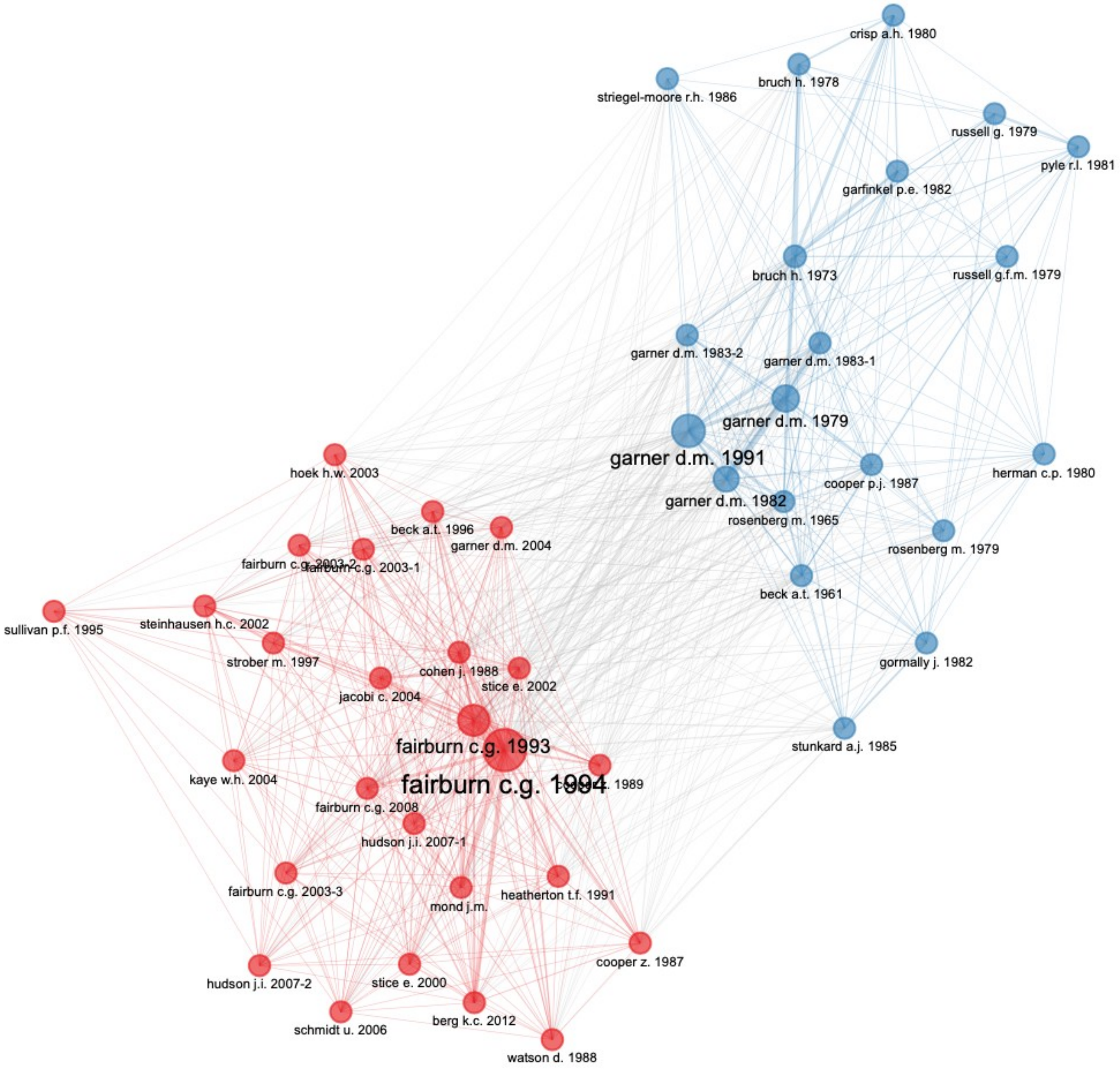

Note. *Analysis performed with *bibliometrix* R package. The figure shows the 46 nodes obtained by the analysis. The Fruchterman-Reingold layout was employed for visualization.
