# Supplementary material for: 40 years of research on eating disorders in domain-specific journals: Bibliometrics, network analysis, and topic modeling
Source: PLoS One. 2022 Dec 15;17(12):e0278981. doi: 10.1371/journal.pone.0278981 (PMC9754234; doi:10.1371/journal.pone.0278981)
Supplement: Attachment — Submitted filename: Response to Reviewers.docx [file pone.0278981.s013.docx]

Responses to **Editor and Reviewers** (in red color)

**Reviewer #1:** No further comments made.

**Reviewer #2:** No further comments made.

**Editor:**

1. The description of a network is not standard. You wrote: . "In network analysis, there are two main components: nodes (vertices) and edges (links between nodes)". Here you are mixing the notion of network with the analysis of networks. Moreover "components" is a technical term in network analysis: better to avoid confusion. So, sraphs or networks are the object studied by network analysis. A network is defined as a a pair of sets: a set of nodes or vertices and a set of edges (link between nodes).

**R1.** Thank you very much for the suggestion. The following change has been made in the manuscript (p. 12 line 10):

*~~In network analysis, there are two main components: nodes (vertices) and edges (links between nodes)~~ Regarding the network, it is defined as a a pair of sets: a set of nodes or vertices and a set of edges (link between nodes)*

2. In describing the methodology you wrote: "More precisely, the Louvain algorithm for community detection [70] was used to create a collaboration network. This algorithm identifies densely connected nodes within the network (i.e., communities) [e.g., 71]". Please note that the collaboration network is not created by the Louvain algorithm. The Louvain algorithm is used for identyfying communities in the collaboration network.

**R2.** Thank you so much for this suggestion. In line with it, the following change has been made (p. 12 line 12):

*More precisely, the Louvain algorithm for community detection [70] was used to ~~create a~~ identify communities within the collaboration network.*
